# Enhanced Recovery After Surgery (ERAS) Protocol for Craniotomy Patients: A Systematic Review

**DOI:** 10.5812/aapm-146811

**Published:** 2024-11-16

**Authors:** Masood Zangi, Mahsa Asadi Anar, Mahdi Amirdosara, Majid Mokhtari, Reza Goharani, Sara Sanei Moghaddam, Omidvar Rezaei, Seyede Hamideh Hashemiyazdi, Mohammadreza Hajiesmaeili

**Affiliations:** 1 Critical Care Quality Improvement Research Center, Loghman Hakim Hospital, Shahid Beheshti University of Medical Sciences, Tehran, Iran; 2 Student Research Committee, School of Medicine, Shahid Beheshti University of Medical Sciences, Tehran, Iran; 3 Skull Base Research Center, Loghman Hakim Hospital, Shahid Beheshti University of Medical Sciences, Tehran, Iran; 4 Department of Anesthesiology, Shahid Madani Hospital, Alborz University of Medical Sciences, Alborz, Iran

**Keywords:** ERAS, Craniotomy, Hospitalization, Neurosurgery

## Abstract

**Context:**

The enhanced recovery after surgery (ERAS) protocol is a multidisciplinary approach aimed at improving surgical outcomes, reducing complications, minimizing hospital stays, and lowering healthcare costs.

**Objectives:**

This study assesses the impact of the ERAS protocol on elective craniotomies, a routine procedure in neurosurgery.

**Methods:**

A comprehensive search across PubMed, Embase, Scopus, and Web of Science identified 562 articles. Following strict screening criteria, 54 studies were reviewed, and ultimately 10 studies meeting the inclusion criteria were selected for detailed analysis.

**Results:**

The review encompassed ten studies [one prospective, one systematic review, and eight randomized controlled trials (RCTs)] published between 2016 and 2023. Key components of the ERAS protocol included preoperative counseling, high-protein intestinal nutrition, preoperative fasting while avoiding carbohydrate intake within 2 hours of surgery, standardized anesthetic and analgesic regimens, and early postoperative initiation of enteral feeding. Postoperative outcomes showed fewer complications, early mobilization, and notably shorter hospital stays, all of which contributed to improved patient recovery.

**Conclusions:**

This review demonstrates that the ERAS protocol, when applied to elective craniotomies, is effective in enhancing postoperative recovery, improving functional outcomes, and reducing hospitalization duration.

## 1. Context

The primary objective of any major surgical procedure is to expedite patient recovery and ensure a return to normal activities without increasing complication rates. Craniotomy is a common procedure in neurosurgery, performed for various reasons such as removing or biopsying brain lesions, operating on intracranial blood vessels, treating epilepsy, and managing trauma. From 2004 to 2007, approximately 70,849 craniotomies were conducted annually in the United States for tumors, 2,237 for vascular procedures, and 56,405 for other reasons (1). Craniotomy, however, is often associated with significant physiological, emotional, and psychological stress, which can increase the risk of cerebrovascular and cardiac complications, impair nutritional absorption, and delay patient recovery. Common postoperative complications include pain, nausea, vomiting, neurological deterioration, and unstable blood pressure, all of which can impede the healing process (2-4).

In 1997, Kehlet and Wilmore introduced the enhanced recovery after surgery (ERAS) protocol to reduce postoperative complications, enhance surgical outcomes, and lower healthcare costs. The ERAS protocol involves a multidisciplinary team—including a surgeon, anesthesiologist, nurse, physiotherapist, and nutritionist—who collaborate to optimize perioperative care for surgical patients. This approach is based on evidence-based practices, covering interventions both before and after surgery. Widely adopted globally, ERAS has led to improved surgical quality, reduced postoperative complications, decreased length of stay (LOS), enhanced patient satisfaction, and reduced healthcare expenditures (5-7). 

The ERAS Association, established in 2010, aims to further enhance postoperative recovery through research, education, and evidence-based practices (8). The ERAS approach has been effectively implemented across numerous surgical specialties, significantly improving recovery times in planned surgeries. The ERAS Association released its first clinical guideline for colorectal surgery in 2012, featuring 24 sections. Since then, the Association has endorsed clinical guidelines for various procedures, including colon and rectal resections, pancreas and duodenum resections, liver resections, gastric and esophageal resections, anesthesia protocols, gynecological surgeries, cystectomy, bariatric surgery, head and neck cancer surgeries, breast reconstruction, joint replacements, and thoracic surgeries (9).

Enhanced recovery after surgery protocols classify essential care elements based on preoperative, intraoperative, and postoperative stages. Pre-operative procedures prepare patients for surgery by administering medication, evaluating risks, and providing patient education. Intra-operative programs focus on reducing surgical stress, utilizing suitable anesthetic techniques, administering local anesthesia, implementing multimodal pain treatment, and minimizing surgical invasiveness. Post-operative therapies aim to accelerate patients' recovery and let them resume their regular diet and daily activities. This entails quickly assessing and managing pain to promote early mobility and utilizing various pain management strategies (10).

Diverse surgical techniques have included distinct ERAS regimens (11-15). Enhanced recovery after surgery protocols, namely craniotomy, have not yet been introduced in neurosurgery. The utilization of ERAS in neurosurgery is a recent development. Introducing ERAS techniques in craniotomies can significantly influence postoperative care (15). Neurosurgery has substantial risks of complications and death, which can dramatically increase with insufficient post-operative care. A precise and balanced method is necessary to improve a patient's surgical recovery process significantly (16). 

Advancements in neurosurgery globally in recent years have resulted in quicker recovery times and reduced hospital stays. However, limited research exists on the use of a tight regimen like ERAS in craniotomy surgery.

## 2. Objectives

In this study, we aimed to conduct a systematic review of randomized controlled trials (RCTs) to assess the impact of ERAS protocols and traditional perioperative care on postoperative outcomes in craniotomy patients.

## 3. Data Sources

### 3.1. Search Strategy and Data Extraction 

This review examines the ERAS protocol for craniotomy, focusing on literature from 2014 to 2023. Relevant studies were identified in scientific journals and databases (Web of Science, Embase, PubMed, Scopus) through a systematic search using keywords such as "Enhanced Recovery," "ERAS," "Craniotomy," and possible combinations, as well as each of the 22 main terms related to ERAS and craniotomy and their combinations (17). Additionally, references from selected articles were screened to locate further relevant studies, prioritizing high-quality studies like RCTs. Two independent reviewers used the RAYAAN tool for systematic reviews to carry out study selection, with a third reviewer involved in resolving conflicts and ensuring consensus. Prospective studies were included when no higher evidence level was available.

### 3.2. Inclusion/Exclusion Criteria and Data Collection 

Inclusion criteria encompassed studies evaluating ERAS in patients undergoing elective craniotomy, those assessing patients under the ERAS protocol as defined by at least one of the two items on the ERAS association recovery checklist or following Hagan et al.'s recommendations (9, 15), and studies confirming that patients provided informed consent. Exclusion criteria included studies involving emergency surgery patients, those with preoperative consciousness disorders, or individuals with conditions (such as pregnancy) or diseases potentially impacting postoperative recovery. Studies that did not utilize ERAS protocols for assessing recovery, involved patients under 18 years of age, or were non-English language studies were also excluded.

**Figure 1.**
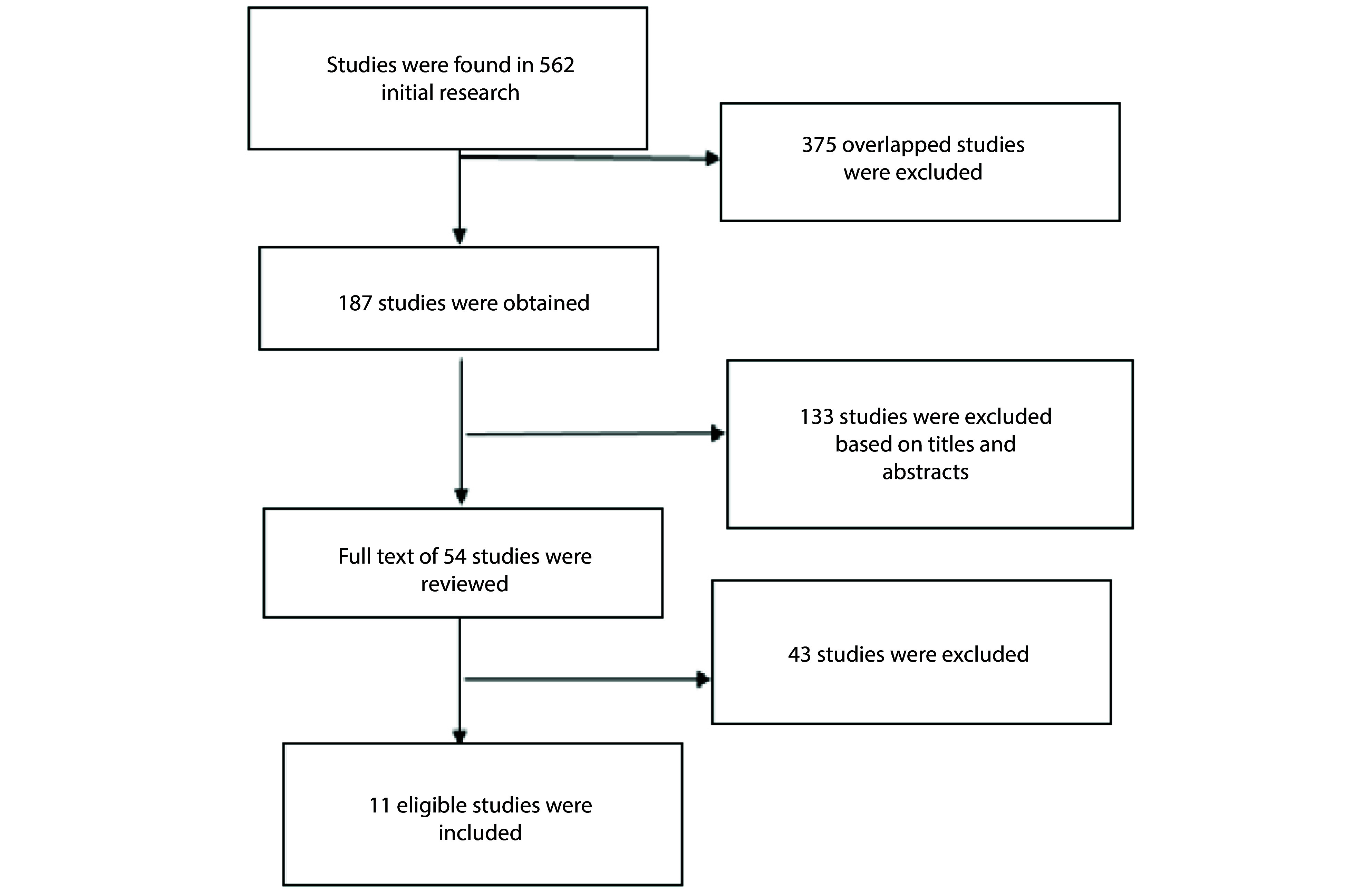
Flowchart of study selection

Firstly, two researchers compiled a list of titles and abstracts from the included papers and assessed them to select relevant documents. The chosen studies were then independently reviewed. Any disagreements between the two researchers were resolved by a third expert. A total of 562 articles were identified in the initial search (Figure 1). After removing duplicates, 187 publications with potential relevance were retained, and their abstracts were reviewed according to specific inclusion and exclusion criteria. Of these, 133 publications were excluded, leaving 54 full-text articles that were evaluated in detail. Ten studies ultimately met the inclusion criteria for the review.

The selected articles underwent a thorough assessment, and data were entered into a specially designed data extraction form. The items reviewed included the study's objective, field of study, sample selection process, inclusion and exclusion criteria, sample size, ethical considerations, ERAS elements examined, postoperative complications, and hospital LOS. This study was approved by the Iranian National Committee for Ethics in Medical Sciences (ethics code: IR.SBMU.RETECH.REC.1402.654).

## 4. Results

Following the elimination of duplicates and irrelevant studies, 11 publications were selected for examination, aligning with the study objectives (Figure 1). This review includes one prospective study, one retrospective review, one systematic review, and eight RCTs. The studies discussed are detailed in Appendix 1 in Supplementary File.

In 2016, Hagan et al. conducted a comprehensive systematic review of data related to oncological craniotomy. They examined ERAS protocol components from various surgical specialties to determine the best evidence-based principles for elective craniotomy patients. This study identified 17 essential components of the ERAS protocol specifically tailored for elective craniotomy. The relevance and recommendations for the 17 ERAS components in craniotomy are outlined in Appendix 1 in Supplementary File. Hagan et al. emphasized the importance of preoperative education and counseling to inform patients about surgical goals and procedures. They recommended that patients abstain from smoking and alcohol for at least one month before surgery and consume high-carbohydrate foods up to two hours before the procedure (15).

To prevent thrombosis, they advocated using pneumatic and graded compression stockings during surgery, alongside prophylactic cefazolin injections for the general population and MRSA prophylaxis one hour before incision to minimize infection risks. Scalp blocks and local anesthetic infiltration along the incision were recommended to reduce postoperative opioid requirements. For pain management, they suggested non-opioid analgesics such as tramadol, gabapentin, pregabalin, and NSAIDs to minimize adverse effects commonly associated with opioid use in craniotomy patients.

Additional recommendations included maintaining normothermia, removing urinary catheters by the first postoperative day, carefully balancing fluids, initiating early enteral feeding, and promoting early mobilization post-surgery. Postoperative nausea and vomiting (PONV), which affect nearly 50% of patients after craniotomy, could be managed by using dexamethasone and serotonin antagonists for prophylaxis.

These recommendations aimed to support rehabilitation and encourage early discharge, though Hagan et al. called for further research to strengthen the evidence base for these ERAS guidelines in craniotomy patients (15). In their RCT at a medical hospital in Xi'an, China, Wang et al. evaluated the safety and efficacy of an ERAS neurosurgical protocol for elective craniotomies. Conducted between October 2016 and May 2017, the study involved 140 participants undergoing elective craniotomy, with 70 assigned to the ERAS group and 70 to the control group. Both groups had comparable discharge criteria, with the ERAS group experiencing shortened hospital stays, earlier catheter removal, and quicker initiation of solid foods. Pain management in the ERAS group relied on oral analgesics, which kept pain levels mild and brief, while providing adequate intestinal nutrition, ensuring mobility restoration, and addressing patients' concerns to enhance satisfaction. The study revealed a statistically significant reduction in hospital stay for the ERAS group, demonstrating faster recovery post-surgery without added risk of complications, thus underscoring the advantages of the ERAS protocol over conventional care for elective craniotomy (18).

In another RCT, Liu et al. (19) examined patient satisfaction with the ERAS protocol post-elective craniotomy at both discharge and a 30-day follow-up. Conducted in China from October 2016 to July 2017, the study included 140 patients undergoing elective craniotomy for brain lesions. Patients were randomly assigned to either the ERAS group, adhering to ERAS-compliant neurosurgical care, or a control group receiving conventional postoperative care, with 70 patients in each group. A standardized questionnaire assessed patient satisfaction at discharge.

Thirty days after enrollment in the ERAS program, a qualitative telephone interview assessed patients' experiences. Findings indicated that average patient satisfaction in the ERAS group post-discharge was significantly higher than in the control group (92.2 ± 4.3 vs. 86.8 ± 7.4). Key factors influencing overall satisfaction included age, use of skin resorption sutures, Visual Analog Scale (VAS) pain scores, postoperative LOS, and incidence of PONV. This study supported the ERAS protocol's role in reducing postoperative hospital stays in craniotomy patients without increasing complication rates (19).

Elayat et al. (20) conducted a non-RCT to evaluate the efficacy of the ERAS protocol in routine care following elective craniotomy. Seventy patients were assigned to either the ERAS (n = 70) or control (n = 70) group. Patients in the ERAS group received care consistent with Hagan et al.'s guidelines (15), covering preoperative, intraoperative, and postoperative stages, while the control group adhered to traditional measures and care protocols. Outcomes compared included ICU LOS, ICU pain scores, opioid requirements, glycemic control, and overall hospital stay duration. The results revealed a significant decrease in the percentage of patients requiring ICU stays longer than 48 hours in the ERAS group (40.6% vs. 65.7%). The ERAS group also experienced less pain, reduced postoperative opioid use, better blood sugar management, and faster mobilization, although total hospital stay duration was similar across both groups (20).

Qu et al. (21) conducted a RCT to assess the effects of ERAS on pain relief and recovery following elective craniotomy. The study involved 129 patients undergoing craniotomy in China, divided into two groups: The ERAS group (n = 64) and the control group (n = 65). Following Hagan et al.’s recommendations (15), patients in the ERAS group received care based on the ERAS protocol, while the control group received standard postoperative care. The Oral Numerical Rating Scale (NRS) measured postoperative pain, and scores were compared between the groups. Results showed that on the first postoperative day, patients in the ERAS group experienced significantly lower pain levels compared to the control group (P < 0.05). Additionally, the ERAS group had shorter hospital stays and lower medical costs. Pain scores were also significantly lower in the ERAS group on the second and third postoperative days. This study concluded that implementing the ERAS protocol in elective craniotomy significantly reduces postoperative pain and enhances recovery, leading to earlier discharge compared to traditional care (21).

Lu et al. (22) conducted a RCT to examine the impact of ERAS on PONV following craniotomy. The study evaluated 105 patients undergoing infratentorial craniotomy, with 64 patients in the ERAS group and 55 in the control group. While the control group received standard postoperative care, the ERAS group followed the ERAS protocol developed by Hagan et al. (15). Outcomes compared between the groups included vomiting frequency, nausea intensity, antiemetic use within the first 72 hours post-surgery, postoperative anxiety, sleep quality, and other postoperative issues. The ERAS group experienced significantly lower rates of vomiting within 72 hours post-craniotomy compared to the control group. More patients in the ERAS group reported mild nausea levels. Additionally, the ERAS group had better outcomes regarding postoperative anxiety (P = 0.01) and sleep quality (P = 0.03) than the control group. This study concluded that ERAS in craniotomy enhances sleep quality and reduces nausea, vomiting, and anxiety without increasing postoperative complications, facilitating a quicker functional recovery (22).

Chen et al. conducted a prospective study to assess the application of the ERAS protocol in elective awake craniotomies. This study, conducted over 16 months from September 2017 to December 2018 at Jinan Hospital, included 20 patients with an average age of 49.5. Data was collected on demographics, health conditions, anesthesia history, intraoperative blood pressure, heart rate, blood gas analysis, perioperative complications, and length of postoperative hospital stay. Among the participants, 20% experienced hypertension, 5% had hypotension, and 5% experienced intraoperative bradycardia. There were no significant intraoperative changes in lactic acid levels, blood glucose, heart rate, or mean arterial pressure. Postoperatively, 5% of patients experienced seizures, 15% reported discomfort, and 10% had nausea or vomiting. The average postoperative hospital stay was 9.5 days, with an ICU stay averaging one day. No cases of 30-day readmission or reoperation were recorded. The findings by Chen et al. indicate that the ERAS protocol resulted in minimal complications and an appropriate duration of stay in both the hospital and ICU (23).

In another study, a RCT was conducted at Xiangya Hospital between January 2019 and June 2020, involving 151 patients undergoing elective craniotomy. Patients were divided into ERAS and control groups. While the control group received standard care, the ERAS group was provided with evidence-based systematic improvement strategies. Outcomes compared between the groups included postoperative LOS, hospitalization costs, 30-day readmission rates, postoperative complications, pain levels, ICU stay duration, time to solid oral feeding, and functional recovery rates. Results indicated that patients in the ERAS group experienced a significantly shorter hospital stay (P < 0.0001), reduced hospital expenses (P < 0.0001), and a 9.2% lower incidence of PONV (P = 0.003) compared to the control group. Patients following the ERAS protocol reported less moderate-to-severe postoperative pain, shorter pain durations, and faster functional recovery (P < 0.001). The findings underscore the effectiveness of the ERAS approach in elective craniotomies, with marked reductions in medical expenses, LOS, and postoperative complications compared to standard postoperative care (24).

Yan et al. conducted a RCT to investigate body composition changes before and after surgery in patients undergoing elective craniotomy, focusing on the effect of the ERAS program on nutritional status—a crucial factor for recovery and functional improvement. From October 2016 to May 2017, 140 patients scheduled for elective craniotomy were randomly assigned to two groups: An ERAS group following the ERAS protocol and a control group receiving standard care. Bioelectrical impedance analysis was used to assess body composition changes in both groups. The study found that patients in the ERAS group maintained a more stable metabolic and nutritional status postoperatively than those in the control group. The hospital stay averaged 10 days in the ERAS group compared to 13 days in the control group, with an average postoperative hospital stay of 4 days versus 7 days, respectively (25).

Wu et al. performed a RCT to evaluate the safety and efficacy of the ERAS protocol in perioperative care for patients with supratentorial tumors. A total of 151 patients were randomly assigned to either a control group (n = 75) receiving conventional neurosurgical care or an ERAS group (n = 76) implementing the ERAS regimen. Conducted at Ningbo Hospital from June 2018 to August 2019, the study analyzed surgical complications, hospitalization duration, timing of initial meal, catheter removal, patient mobility, and postoperative recovery quality in both groups. Results indicated that postoperative feeding, catheter removal, and mobilization occurred earlier in the ERAS group than in the control group. The ERAS group had a significantly shorter LOS, averaging 8 days compared to 11 days in the control group (P < 0.001). Additionally, the ERAS group showed lower readmission and reoperation rates, while postoperative complication rates were similar between the groups. The findings suggest that the ERAS protocol is a safe and effective approach for patients with supratentorial tumors, reducing surgical stress, expediting recovery, and decreasing hospital stay (26).

McLaughlin et al. conducted a retrospective study on patients with trigeminal neuralgia or hemifacial spasm who had undergone Microvascular Decompression (MVD). The study analyzed two patient groups: Group 1 (20 patients) underwent surgery before the ERAS protocol's introduction in 2008 - 2009, while group 2 (29 patients) received surgery during the ERAS protocol's implementation in 2011 - 2012. Both groups experienced significant symptom reduction with no reported deaths. The group with the improvement strategy saw reductions in operation time, postoperative hospital stay, costs, and readmissions (27). The studies reviewed highlighted variations in surgical techniques and tumor locations. Due to the lack of a standardized ERAS protocol for neurosurgery, the specific ERAS elements applied varied across the studies.

## 5. Discussion

This study examined the effectiveness of the ERAS program in elective craniotomy. We reviewed 11 research studies assessing the impact of the ERAS procedure on patients undergoing craniotomy. Enhanced recovery after surgery procedures are widely utilized in the postoperative period across multiple surgical specialties, including colorectal surgery, urology, and orthopedics. These interventions have consistently resulted in reduced hospital stays, improved functionality, and decreased complications. However, research on the application of the ERAS technique for elective craniotomy remains limited (14, 28-30).

Hagan et al. conducted an extensive review of data on oncological craniotomy, proposing essential components for the ERAS approach. These components were derived from elective craniotomy patient needs and related aspects from other surgical disciplines that are applicable to neurosurgery patients. The authors suggested that further research is necessary to strengthen the quality of evidence supporting ERAS in this context (15). Wang et al. provided robust evidence for the effectiveness of ERAS in neurosurgery through a RCT, supporting its application in elective craniotomy (18).

The ERAS protocol includes components such as preoperative counseling, high-protein preoperative intestinal nutrition, fasting, carbohydrate intake up to two hours before surgery, standard anesthetic and analgesic treatments, and the early initiation of postoperative feeding. While ERAS principles are broadly effective, some elements may require adaptation for craniotomy surgeries. Innovations such as scalp blocks and minimally invasive surgery (MAS) are crucial for accelerating recovery post-treatment (10). Enhanced recovery after surgery protocols play a vital role in craniotomy surgeries, significantly influencing the length of hospital stay, postoperative pain levels, and functional recovery outcomes.

The ERAS approach emphasizes detailed and meticulous pre- and post-operative care, requiring collaboration among neurosurgeons, anesthesiologists, surgical assistants, operating room nurses, neurophysiologists, nutritionists, and family support to optimize recovery (31-34). Wang et al.'s study supports the safe and effective use of oral carbohydrates two hours before surgery for certain craniotomy patients (18).

Minimally invasive surgery aims to limit surgical trauma, thereby reducing postoperative discomfort, enhancing mobility, and minimizing inpatient stays and associated complications (35). Minimally invasive surgery is favored by patients and healthcare providers due to its capacity to expedite healing and facilitate an earlier return to daily activities. The reduction in tissue dissection is a key factor contributing to decreased postoperative pain in MAS (36). The focus of ERAS in craniotomy includes improving aesthetic outcomes, reducing discomfort, facilitating early discharge, using less invasive techniques, and incorporating endoscopy where appropriate (37).

Respiratory management is a critical component of the ERAS protocol and is continuously monitored throughout the surgical process. Craniotomy patients face a substantial risk of thromboembolic events, with incidence rates reaching up to 30%. Utilizing both mechanical and chemoprophylaxis methods can reduce the incidence of thromboembolic events to below 1% in these patients (38). In craniotomy, mechanical prophylaxis is generally preferred over pharmacological methods due to the increased risk of bleeding. Mechanical prophylaxis includes using calibrated compression stockings and pneumatic intermittent compression devices to mitigate venous thromboembolism (VTE) risk (16). Chemoprophylaxis is recommended for high-risk patients, such as those with prolonged immobility, a history of thromboembolic disease, varicose veins, and significant neurological impairments (38).

Postoperative pain is a considerable stressor, potentially leading to extended bed rest, delayed discharge, impaired recovery, and a reduced quality of life for patients (39). Effective pain management is, therefore, a key element of the ERAS protocol. Advances in postoperative pain management following craniotomy show that patients often experience moderate to severe pain immediately after the procedure, which may persist for several months (40).

Studies indicate that employing a comprehensive pain management approach within the ERAS protocol can reduce the need for long-acting or high-dose opioids (15). Selecting analgesics that minimize cognitive and orientational effects is particularly crucial in craniotomy procedures. To prevent the delayed detection of significant intracranial pressure, the anesthetic regimen should avoid long-acting opioids due to side effects such as drowsiness, miosis, nausea, and vomiting. Opioids can also impact cerebral blood flow by elevating blood carbon dioxide levels, leading to respiratory complications (41). Intraoperative anesthetics and analgesics, including dexmedetomidine, ketamine, and lidocaine, play an essential role in meeting ERAS criteria post-craniotomy. These medications aid in blood pressure control, reduce inflammation, and limit opioid dependency, though they may potentially impact postoperative cognitive recovery (42, 43).

Hagan et al. (15) identified gabapentin/pregabalin and tramadol as potentially detrimental to analgesia in craniotomy patients within the ERAS framework. The effectiveness of intravenous acetaminophen for craniotomy pain remains uncertain, while COX-2 inhibitors and specific doses of flupirtine show promise for post-craniotomy pain management; however, further studies are needed to confirm their safety and efficacy. Scalp blocks and infiltration techniques are effective in reducing hemodynamic stress, enhancing intraoperative hemodynamic stability, and decreasing postoperative opioid requirements (44). Research supports that regional anesthesia techniques like scalp blocks and infiltration can expedite recovery following craniotomy (45-47) by minimizing the need for narcotics and anesthetics, mitigating the surgical inflammatory response, and consequently reducing hospital stays. These findings align with Wang et al.'s study (24).

Postoperative nausea and vomiting are common symptoms following surgery with multiple potential causes, impacting approximately 47% of patients after craniotomy (48). Effective management of PONV post-craniotomy is essential as it can destabilize intracranial pressure. Serotonin receptor antagonists and dexamethasone are highly recommended for mitigating PONV due to their potent inhibitory effects (16). Additionally, transcutaneous electrical stimulation, a non-pharmacological intervention, has been shown to alleviate nausea, vomiting, and postoperative pain in craniotomy patients (49).

The ERAS protocol significantly reduces PONV in post-craniotomy patients (15, 18), a finding consistent with the results of our comprehensive review (18, 19, 22-24). The ERAS care team includes physicians, nurses, dietitians, rehabilitation specialists, physical therapists, and psychologists, collectively working to elevate the overall quality of patient care. Evaluating ERAS teamwork requires an in-depth analysis to identify gaps in patient care and determine the effectiveness of each ERAS component. Studies show a 70% effectiveness rate with reduced mortality when ERAS protocols are strictly followed (50). Research further indicates that adherence to ERAS guidelines reduces surgical complications, ICU admissions for severe complications, and mortality rates (50, 51).

Enhanced recovery after surgery aims to shorten hospital stays, reduce surgical complications, and enhance patient satisfaction. Implementing ERAS protocols and improving quality standards have also resulted in significant cost savings (52). Therefore, ERAS approaches provide substantial benefits to healthcare providers, administrators, policymakers, patients, and society as a whole (8).

Enhanced recovery after surgery has redefined traditional pre-, intra-, and post-operative care practices, helping to alleviate pre-surgery anxiety and enhancing post-surgery recovery. Additionally, ERAS is a systematic and precise interdisciplinary approach that promotes improved treatment outcomes. Implementing ERAS for craniotomy patients appears promising in achieving intended results, especially through techniques like scalp blocks, non-opioid pain management, and MAS, which slightly differ from the standard ERAS protocol. Using ERAS for craniotomy can improve surgical outcomes, speed up functional recovery, and reduce hospital stay duration. However, further research is necessary to refine ERAS components and improve postoperative results for craniotomy patients.

This review faces certain limitations. Primarily, all RCTs included in the study are likely susceptible to bias. In this context, fully concealing participant and clinical staff identities poses a significant challenge. The studies analyzed also demonstrated variability in surgical procedures and tumor locations. Due to the absence of a definitive ERAS protocol for neurosurgery, different studies included varied ERAS components. Additionally, limited research and substantial variability in outcomes warrant caution when interpreting findings. Data were insufficient to fully determine clinical outcomes, such as complication and mortality rates. Further research is essential to pinpoint the most beneficial ERAS elements and their therapeutic impact across neurosurgical settings. Rigorous data from multicenter trials are needed to establish ERAS guidelines for perioperative management in post-craniotomy care.

aapm-14-5-146811-s001.pdf

## Data Availability

The data presented in this study are uploaded during submission as a supplementary file and are openly available for readers upon request.
