# Supplementary material for: Enhanced Recovery After Surgery (ERAS) Protocol for Craniotomy Patients: A Systematic Review
Source: Anesth Pain Med. 2024 Nov 16;14(5):e146811. doi: 10.5812/aapm-146811 (PMC11895796; doi:10.5812/aapm-146811)
Supplement: aapm-14-5-146811-s001.pdf [file aapm-14-5-146811-s001.pdf]

Appendix 1

Table1. Guidelines for preoperative care for craniotomy: Enhanced recovery after surgery recommendations (ERAS) (10).

	Item	Summary and recommendations	Evidence level	Recommendation grade
preoperative	Preoperative counseling	Patients should be routinely counseled regarding what to expect from them surgical experience	Low	Strong
	Preoperative smoking and alcohol consumption	Abstinence from both alcohol and smoking for 1 month is recommended when appropriate and feasible	Moderate	Strong for
	Preoperative enteral nutrition and perioperative oral immunonutrition	Enteral nutrition is recommended preoperatively, and immunonutrition may be important for patients with cancer, although larger studies are needed	Enteral nutrition: Moderate Immunonutrition: Moderate	Enteral nutrition: Strong Immunonutrition: Weak
	Preoperative fasting and carbohydrate loading	As carbohydrate beverages are a clear liquid, perioperative carbohydrate loading should be encouraged	Carbohydrate loading: Low	Carbohydrate loading: Strong
	Anti-thrombotic prophylaxis	Graduated compression stockings and intermittent pneumatic compression are advised for craniotomy patients to prevent venous thromboembolism. Avoid using anticoagulants regularly.	High	Strong
	Antimicrobial prophylaxis and skin preparation	Minimize scalp shaving. Routine prophylaxis with cefazolin within 1 hour prior to skin incision is recommended. Patients with MRSA should be treated prophylactically with vancomycin initiated 1 hour prior to skin incision	Scalp shaving: Moderate Antibiotic prophylaxis: High	Scalp shaving: Weak against Antibiotic prophylaxis: Strong
	Scalp blocks	Both scalp infiltration and scalp blocks are recommended	Moderate	Strong

		for craniotomies		
	Anesthetic protocol	The evidence does not support the superiority of short versus longer acting anesthetics or TIVA versus pure inhalation anesthetics in craniotomies	TIVA: High<br>IV lidocaine, ketamine: High<br>Dexmedetomidine: High	TIVA: Weak<br>IV lidocaine, ketamine: Strong against<br>Dexmedetomidine: Weak
Intraoperative	Non-opioid analgesia	The adverse effect profiles of tramadol and gabapentin/pregabalin are not good for craniotomy. Although its effectiveness in treating craniotomy patients has not yet been established, IV acetaminophen has a manageable side effect profile. While more study is conducted to ensure the safety and efficacy of COX-2 inhibitors and flupirtine, there may be a role for limited dosage of these medications.	IV acetaminophen: Moderate<br>Gabapentin/ pregabalin/tramadol: Low<br>COX-2 inhibitors/ flupirtine: Low	IV acetaminophen: Strong<br>Gabapentin/ Pregabalin/tramadol: Weak against<br>COX-2 inhibitors/ flupirtine: Weak
	PONV	Regular use of dexamethasone and serotonin receptor antagonists is recommended. Because of its high cost and limited ability to reduce the need for rescue anti-emetics, aparpepitant is best reserved for patients who have a high risk of PONV. For TEAS, more research is required. Promethazine and scopolamine both have side effects that make them inappropriate as first-line anti-nausea medications	Dexamethasone and serotonin antagonists: High<br>Aprepitant, TEAS: Low<br>Scopolamine, promethazine: Low	Dexamethasone and serotonin antagonists: Strong<br>Aprepitant, TEAS: Weak<br>Scopolamine, promethazine: Weak against
	Minimally invasive craniotomies and endoscopic skull base approaches	Minimally invasive neurosurgery has the potential to improve patient satisfaction and recovery, however there are few randomized controlled trials that demonstrate improved outcomes	Very low	Weak
	Avoiding hypothermia	Measures to prevent hypothermia should be implemented for all elective	High	Strong

		craniotomies		
	Fluid balance	Non-invasive cardiac output monitoring may offer more accurate<br><br>determination of volume status.	low	Strong
Postoperative	Urinary drainage	Bladder catheters should be removed on postoperative day 1 or as early as is<br><br>feasible	Moderate	Strong
	Postoperative artificial nutrition	Postoperative artificial nutrition is not typically needed for these patients, with<br><br>the exception of patients who are in a prolonged comatose state	Moderate	Strong
	Early mobilization	Encourage early mobilization	High	Strong
	Audit	Audit is a valuable tool for assessing impact and encouraging compliance	Moderate	Strong
preoperative	**Item**	**Summary and recommendations**	**Evidence level**	**Recommendation grade**
	Preoperative counseling	Patients should be routinely counseled regarding what to expect from their<br><br>surgical experience	Low	Strong
	Preoperative smoking and<br><br>alcohol consumption	Abstinence from both alcohol and smoking for 1 month is recommended when<br><br>appropriate and feasible	Moderate	Strong for
	Preoperative enteral nutrition<br><br>and perioperative oral<br><br>immunonutrition	Enteral nutrition is recommended preoperatively, and immunonutrition may<br><br>be important for patients with cancer, although larger studies are needed	Enteral nutrition:<br><br>Moderate<br><br>Immunonutrition:<br><br>Moderate	Enteral nutrition: Strong<br><br>Immunonutrition: Weak
	Preoperative fasting and<br><br>carbohydrate	As carbohydrate beverages are a clear liquid, perioperative carbohydrate<br><br>loading should be encouraged	Carbohydrate loading:<br><br>Low	Carbohydrate loading:<br><br>Strong

	loading			
	Anti-thrombotic prophylaxis	For patients with craniotomies, the use of graduated compression stockings and intermittent pneumatic compression is advised in order to prevent venous thromboembolism. It is not advised to take anticoagulants regularly.	High	Strong
	Antimicrobial prophylaxis and skin preparation	Minimize scalp shaving. Routine prophylaxis with cefazolin within 1 hour prior to skin incision is recommended. Patients with MRSA should be treated prophylactically with vancomycin initiated 1 hour prior to skin incision	Scalp shaving: Moderate<br>Antibiotic prophylaxis: High	Scalp shaving: Weak against<br>Antibiotic prophylaxis: Strong
	Scalp blocks	Both scalp infiltration and scalp blocks are recommended for craniotomies	Moderate	Strong
	Anesthetic protocol	The evidence does not support the superiority of short versus longer-acting anesthetics or TIVA versus pure inhalational anesthetics in craniotomies	TIVA: High<br>IV lidocaine, ketamine: High<br>Dexmedetomidine: High	TIVA: Weak<br>IV lidocaine, ketamine: Strong against<br>Dexmedetomidine: Weak
Intraoperative	Non-opioid analgesia	Tramadol and gabapentin/pregabalin have side effects that make them unsuitable for craniotomy. While it has a benign side effect profile, IV acetaminophen has not been shown to be useful in individuals having craniotomy. Flupirtine and COX-2 inhibitors may have limited uses in the future, pending further research verifying their efficacy and safety	IV acetaminophen: Moderate<br>Gabapentin/ pregabalin/tramadol: Low<br>COX-2 inhibitors/ flupirtine: Low	IV acetaminophen: Strong<br>Gabapentin/ Pregabalin/tramadol: Weak against<br>COX-2 inhibitors/ flupirtine: Weak
	PONV	Regular use of dexamethasone and serotonin receptor antagonists is recommended. Because of its high cost and limited ability to reduce the need for rescue anti-emetics, aparpepitant is best reserved for patients	Dexamethasone and serotonin	Dexamethasone and serotonin

		who have a high risk of PONV. Further research is required for TEAS. Promethazine and scopolamine both have side effects that make them inappropriate as first-line anti-nausea medications	antagonists: High<br>Aprepitant, TEAS: Low<br>Scopolamine, promethazine: Low	antagonists: Strong<br>Aprepitant, TEAS: Weak<br>Scopolamine, promethazine: Weak against
	Minimally invasive craniotomies and endoscopic skull base approaches	Minimally invasive neurosurgery offers possibilities for improved patient recovery and satisfaction, but there is a lack of RCT that demonstrates improved outcomes	Very low	Weak
	Avoiding hypothermia	Measures to prevent hypothermia should be implemented for all elective craniotomies	High	Strong
	Fluid balance	Non-invasive cardiac output monitoring may offer more accurate determination of volume status.	low	Strong
Postoperative	Urinary drainage	Bladder catheters should be removed on postoperative day 1 or as early as is feasible	Moderate	Strong
	Postoperative artificial nutrition	Postoperative artificial nutrition is not typically needed for these patients, with the exception of patients who are in a prolonged comatose state	Moderate	Strong
	Early mobilization	Encourage early mobilization	High	Strong
	Audit	Audit is a valuable tool for assessing impact and encouraging compliance	Moderate	Strong
	**Item**	**Summary and recommendations**	**Evidence level**	**Recommendation grade**

preoperative	Preoperative counseling	Patients should be routinely counseled regarding what to expect from their surgical experience	Low	Strong
	Preoperative smoking and alcohol consumption	Abstinence from both alcohol and smoking for 1 month is recommended when appropriate and feasible	Moderate	Strong for
	Preoperative enteral nutrition and perioperative oral immunonutrition	Enteral nutrition is recommended preoperatively, and immunonutrition may be important for patients with cancer, although larger studies are needed	Enteral nutrition: Moderate Immunonutrition: Moderate	Enteral nutrition: Strong Immunonutrition: Weak
	Preoperative fasting and carbohydrate loading	As carbohydrate beverages are a clear liquid, perioperative carbohydrate loading should be encouraged	Carbohydrate loading: Low	Carbohydrate loading: Strong
	Anti-thrombotic prophylaxis	The use of graduated compression stockings and intermittent pneumatic compression is recommended in craniotomy patients to prevent venous thromboembolism. The routine use of anticoagulants is not recommended	High	Strong
	Antimicrobial prophylaxis and skin preparation	Cut back on how often you shave your scalp. Cefazolin should be given as a preventative measure no later than one hour before to the skin incision. Vancomycin should be given to MRSA patients as a preventative measure beginning one hour prior to performing a skin incision	Scalp shaving: Moderate Antibiotic prophylaxis: High	Scalp shaving: Weak against Antibiotic prophylaxis: Strong
	Scalp blocks	Both scalp infiltration and scalp blocks are recommended for craniotomies	Moderate	Strong
	Anesthetic protocol	The evidence does not support the superiority of short versus longer acting anesthetics or TIVA versus pure inhalational anesthetics	TIVA: High IV lidocaine, ketamine: High	TIVA: Weak IV lidocaine, ketamine:

		in craniotomies	Dexmedetomidine: High	Strong against Dexmedetomidine: Weak
Intraoperative	Non-opioid analgesia	Tramadol and gabapentin/pregabalin have side effects that make them unsuitable for craniotomy. Although the effectiveness of IV acetaminophen in the craniotomy group is still unknown, its side effect profile is benign. Flupirtine and COX-2 inhibitors may have limited uses in the future, pending further research verifying their efficacy and safety	IV acetaminophen: Moderate Gabapentin/ pregabalin/tramadol: Low COX-2 inhibitors/ flupirtine: Low	IV acetaminophen: Strong Gabapentin/ Pregabalin/tramadol: Weak against COX-2 inhibitors/ flupirtine: Weak
	PONV	Regular use of dexamethasone and serotonin receptor antagonists is recommended. Because of its high cost and limited ability to reduce the need for rescue anti-emetics, aparpepitant is best reserved for patients who have a high risk of PONV. Further research is required for TEAS. Due to its adverse effects, promethazine and scopolanmine are not recommended as first-line anti-nausea medications	Dexamethasone and serotonin antagonists: High Aprepitant, TEAS: Low Scopolamine, promethazine: Low	Dexamethasone and serotonin antagonists: Strong Aprepitant, TEAS: Weak Scopolamine, promethazine: Weak against
	Minimally invasive craniotomies and endoscopic skull base approaches	Minimally invasive neurosurgery offers possibilities for improved patient recovery and satisfaction, but there is a lack of RCT that demonstrates improved outcomes	Very low	Weak
	Avoiding hypothermia	Measures to prevent hypothermia should be implemented for all elective craniotomies	High	Strong
	Fluid balance	Non-invasive cardiac output monitoring may offer more accurate	low	Strong

		determination of volume status.		
Postoperative	Urinary drainage	Bladder catheters should be removed on postoperative day 1 or as early as is feasible	Moderate	Strong
	Postoperative artificial nutrition	Postoperative artificial nourishment is generally unnecessary for these patients, except for those in a prolonged vegetative condition.	Moderate	Strong
	Early mobilization	Encourage early mobilization	High	Strong
	Audit	An audit is a great instrument for evaluating impact and promoting adherence.	Moderate	Strong

Table2. Outcome summary of the ERAS elements and protocols reported in the included studies.

**Article**	**year**	**Sample size**	**ERAS protocol**	**LOS**	**LOS ICU**	**Pain**	**PONV**	**Patient Satisfaction**	**Conclusion**
Hagan, Bhavsar [15]	2016		See Table 1.						ERAS therapies for oncological craniotomies can enhance patient outcomes, speed up functional recovery, reduce hospital stays, and facilitate the initiation of adjuvant chemotherapy and radiation therapy in a more efficient manner, all of which are indicators of long-term survival.
Wang, Liu [18]	2017	Eras:70<br>Control: 70	The protocol consists of three main parts: preoperative care, surgical and	Eras:4 days<br>Control: 7		Eras: 79% Mild<br>19% Moderate<br>Control: 33% Mild	There was no significant differen	Eras: 92 %<br>Control:	Implementing ERAS is associated with a significant reduction in the postoperative hospital stay and an

			anesthetic procedures, and postoperative care. Preoperative care includes counseling, functional status evaluation, smoking cessation, mental state assessment, antithrombotic therapy, and managing postoperative nausea and vomiting	days<br><br>(p <0.0001)		42% Moderate<br><br>(p <0.0001)	ce between the two groups (p = 0.115)	86%<br><br>(p=0.0001)	acceleration in recovery without increasing complication rates related to elective craniotomy.
[19]	2018	Eras:70<br><br>Control: 70	Preoperative procedures involve patient evaluation, counseling, functional assessment, nutritional evaluation, and cessation of smoking and alcohol use. Intraoperative techniques include minimally invasive surgery, local anesthesia, pain relief, and fluid management. Postoperative care includes pain management and early walking	Eras: 45.7% ≤4<br><br>Control: 10% ≤4<br><br>(p <0.0001)		Eras: 78.6% Mild<br><br>18.6% Moderate<br><br>Control: 32.9% Mild<br><br>60% Moderate<br><br>(p <0.0001)	Eras: 85.7% Mild<br><br>10% Moderate<br><br>Control: 71.4% Mild<br><br>21.4% Moderate<br><br>(p = 0.115)	Eras: 92.2 %<br><br>Control: 86.8%<br><br>(p=0.0001)	Measures that include decreasing the PONV VAS score, incorporating absorbable ssuture,sture and shortening LOS seem to increase patient satisfaction in a neurosurgicalprogramgramme
	2021	Eras:35<br><br>Control: 35	See Table 1.		patients staying in ICU/HDU for more than 48 h was	Eras: 80% Mild<br><br>Control: 54% Mild<br><br>(p = 0.02)			The study significantly reduced patients requiring ICU/ HDU stay > 48 hours. better pain and glycemic control in the

					lesser in the ERAS group than in the Control group. (p = 0.003)				postoperative period may have contributed to a decreased stay.
Qu L (21)	2020	Eras:64<br><br>Control:65	The protocol consists of three main parts: preoperative care, surgical and anesthetic procedures, and postoperative care. Preoperative care includes counseling, functional status assessment, mental state evaluation, antithrombotic therapy, and managing postoperative nausea and vomiting	Eras:4 days<br><br>Control: 7 days<br><br>(P < 0.001)		Eras: 68.8% Mild<br><br>28.1% Moderate<br><br>Control: 35.4% Mild<br><br>60% Moderate<br><br>(p <0.001)			Implementing a neurosurgical ERAS protocol for elective craniotomy patients has significant benefits in alleviating postoperative pain and enhancing recovery, leading to early discharge after surgery compared to conventional care.
Lu D (22)	2020	Eras: 50<br><br>Control: 55	See Table 1.				Eras:62 %<br><br>Control: 40%<br><br>(p = 0.03)		Implementing ERAS protocol in craniotomy patients could attenuate postoperative anxiety, improve sleep quality, and reduce the incidence of PONV without increasing the rate of postoperative complications.
Chen Y (23)	2021	Eras: 20	The surgical procedure involves comprehensive preoperative counseling, intensive protocol-	Eras:9.5 days	Eras:1 day	Eras:15%	Eras:10 %		Applying an ERANS protocol was feasible, associated with a low incidence of complications, and acceptable intensive

			based care, rapid intravenous fluid delivery, ultrasound-guided nerve block, continuous anesthetic monitoring, tailored fluid treatment, and strict postoperative diet and exercise						care unit and postoperative hospital lengths of stay
Wang L (24)	2022	Eras: 76<br>Control: 75	The preoperative bundle includes pre-surgical counseling, fasting, pulmonary function testing, scalp nerve blocks, fluid control, intraoperative monitoring, thermoregulation, and a surgical aftercare kit for pain management	Eras:3 days<br>Control: 4 days<br>(P < 0.0001)	Eras:0 days<br>Control: 0 days	NRS score (2 h)<br>Eras: 2.5<br>Control: 2.9<br>(p< 0.005)<br>NRS score (24 h)<br>Eras: 1.5<br>Control: 2.6<br>(P < 0.0001)	Eras:9.2 %<br>Control: 28%<br>(p = 0.003)		Implementing an enhanced recovery after elective craniotomy protocol had significant benefits over conventional perioperative management. It was associated with significantly reducing postoperative length of stay, medical cost, and postoperative complications.
Yan J (25)	2019	Eras: 70<br>Control: 70	The protocol consists of three main parts: preoperative management, surgical and anesthetic	Eras:4 days<br>Control: 7 days					Patients undergoing elective craniotomy showed various degrees of nutritional injuries, and ERAS could alleviate nutritional injuries caused by the operation.

			management, and postoperative management. Preoperative management involves counseling, functional assessment, and medication, surgical management involves surgery, and postoperative management involves dietary needs and mobilizati						

			on						
Wu J (26)	2021	Eras: 76<br><br>Control: 75	Preoperative treatment include preoperative assessment and ERAS teaching. Preoperative airway management, deep vein thrombosis prophylaxis, and preoperative gastrointestinal care. Preoperative medical procedures and skin cleansing<br><br>Postoperative care includes monitoring, gastrointestinal management, prevention of deep vein thrombosis, tube care, pain management, and early mobilization.	Eras:8 days<br><br>Control: 11 days<br><br>(P <0.001)					ERAS can reduce after-surgical stress and accelerate postoperative recovery, shorten hospital stays, and reduce hospital costs.
McLaughlin (27)	2014	Group 2 intervention: 29<br><br>Group 1 Control: 20	The text provides an overview of the postoperative healing process, including the expected hospital stay and objectives for fluid intake, nutrition, bowel function, mobility, pain management, and a 1-2 day hospital stay. It also discusses post-surgery tasks like communication template creation, discharge	intervention: 3.05 days<br><br>Control: 2.59 days<br><br>(p = 0.041)	intervention: 26 hrs<br><br>Control: 33 hrs	TN pts: control: 11 vs. 11 no pain; intervention: 13 v.s 18 no painfour 4 signifidecreasesron ease, 1 moderate improvement			Comprehensive implementation of improvement processes throughout the continuum of care resulted in improved global outcomes and greater value of delivered care.

			preparations, and standardization of care procedures						

PONV = postoperative nausea and vomiting, ERAS = enhanced recovery after surgery, LOS = length of stay
